# Landing maneuvers of houseflies on vertical and inverted surfaces

**DOI:** 10.1371/journal.pone.0219861

**Published:** 2019-08-14

**Authors:** Sujay Balebail, Sathish K. Raja, Sanjay P. Sane

**Affiliations:** National Centre for Biological Sciences (NCBS), Tata Institute of Fundamental Research, Bangalore, India; Brown University, UNITED STATES

## Abstract

Landing maneuvers of flies are complex behaviors which can be conceptually decomposed into sequences of modular actions, including body-deceleration, leg-extension, and body rotations. These behavioral ‘modules’ must be coordinated to ensure well-controlled landing. The composite nature of these behaviors induces kinematic variability, making it difficult to identify the central rules that govern landing. Many previous studies have relied on tethered preparations to study landing behaviors, but tethering induces experimental artefacts by forcing some behaviors to operate in open-feedback control loop while others remain closed-loop. On the other hand, it is harder for the experimenter to control the stimuli experienced by freely-flying insects. One approach towards understanding general mechanisms of landing is to determine the common elements of their kinematics on surfaces of different orientations. We conducted a series of experiments in which the houseflies, *Musca domestica*, were lured to land on vertical (*wall landings*) or inverted (*ceiling landings*) substrates, while their flight was recorded with multiple high-speed cameras. We observed that, in both cases, well-controlled landings occurred when the distance at which flies initiated deceleration was proportional to flight velocity component in the direction of substrate. The ratio of substrate distance and velocity at onset of deceleration (*tau*) was conserved, despite substantial differences in mechanics of vertical vs. ceiling landings. When these conditions were not satisfied, their landing performance was compromised, causing their heads to collide into the substrate. Unlike body-deceleration, leg-extension in flies was independent of substrate distance or approach velocity. Thus, the robust reflexive visual initiation of deceleration is independent of substrate orientation, and combines with a more variable initiation of leg-extension which depends on surface orientation. Together, these combinations of behaviors enable flies to land in a versatile manner on substrates of various orientations.

## Introduction

In the natural world, the substrates on which flying insects land are of diverse orientations, textures and flexibility [1–3]. From the controls perspective, smooth landing on such diverse substrates requires insects to rapidly sense and adaptively respond to the approaching objects. While landing, insects typically decelerate [4–9], extend their legs [1–3,6,8,10,11], and align their body parallel to the substrate [2,12]. Previous studies have suggested that landing behaviors can be subdivided into these distinct, independently-activated ‘modular’ behaviors [8] that must be mutually coordinated by their nervous system.

What strategies underlie the versatile landing abilities of insects? Landing strategies must ensure that insects have sufficient time to decelerate, thereby avoiding impact injuries. The sensory cues eliciting onset of deceleration have been studied in freely-flying houseflies *Musca domestica* [9] and fruit flies *Drosophila melanogaster* [8]. An important parameter in these studies was the parameter *tau*, conventionally defined as the ratio of distance of insect from the substrate (henceforth, *substrate distance*) and velocity in direction of object (e.g. [13] and associated discussion by Kalmus; also [4]). The value of *tau* represents time-to-collision as the insect flies towards the substrate. [9]showed that houseflies approaching spherical substrates initiated deceleration when *tau* fell below a threshold value. Thus, flies approaching substrates at higher velocities initiated deceleration proportionately further from the object i.e. at constant *tau*.

Landing flies primarily rely on the optic flow over their retina to ascertain the speed of an approaching substrate. Accounting for this, [9]proposed the Relative Retinal Expansion Velocity (RREV) model, in which flies initiate deceleration at a critical value of the ratio of retinal expansion velocity to retinal size of an object. For small landing objects, *tau* is a first-order approximation of RREV [13]. To explain their data on landings in *Drosophila melanogaster*, [8] proposed a Retinal Size-Dependent Expansion Threshold (RSDET) Model which specifically addressed the onset of deceleration as flies approached cylindrical posts. According to this model, deceleration was initiated at threshold values of retinal size-dependent expansion of the object on fly retina. Their instantaneous approach speed was proportional to the logarithm of angular size of the post on fly retina. How rapidly flies cross the retinal size threshold depends on their approach speed, but not the physical dimensions of the substrate. Thus, slowly expanding small objects are as likely to trigger the onset of deceleration as rapidly expanding large objects. Similarly, flies flying further away, but faster, would initiate deceleration, as would flies that are closer but slower. In most respects, the RSDET model resembles RREV or *tau*-estimation models.

While landing, animals control their rate of deceleration to achieve smooth touchdown. Hummingbirds [5] and pigeons [6], control deceleration by maintaining a constant rate of change of *tau* between 0.5 and 1. Honeybees, on the other hand, maintain fixed values of *tau* after initiating deceleration. Thus, flight velocity normal to the substrate reduces linearly with substrate distance [4,7]. Freely-flying insects also extend their legs before contacting the substrate [1–3,6,8,11]. The onset of deceleration and leg-extension thus are the key variables of interest for studies on landing.

The rules governing onset of leg-extension response in free-flight have been previously investigated [1,3,4,6,8,10]. For instance, pigeons approaching a landing perch initiate leg-extension at a fixed *tau* [6]. Honeybees [1] and bumblebees [3] approaching planar surfaces hover and extend their legs at constant distance from the substrate, but irrespective of its inclination. For *Drosophila melanogaster* approaching a cylindrical post, onset of leg-extension is independent of approach velocity, but depends on threshold distance from post, or threshold angle subtended on their retina [8].

It is thought that body-deceleration and leg-extension behaviors can be independently activated. For example, front-to-back optic flow elicits a leg-extension response in tethered insects [10,14–24] although there is no physical deceleration or change in body pitch. This behavior is analogous to leg-extension in freely-flying insects before landing. In tethered houseflies, the time course of leg-extension is constant regardless of the nature of releasing stimulus. However, the latency of leg-extension response depends on optic flow[14], and on the size, velocity, and contrast of looming stimuli [10,19,20]. Besides extending their legs, tethered flies also reduce their thrust in response to a looming stimulus, the onset of which is correlated to leg-extension [19].

Despite the extensive research on landing responses, several questions relating to mutual coordination between leg-extension and body-deceleration remain largely unanswered. What cues elicit the initiation of both modules? Are these modules inter-dependent or independent? Does landing behavior change with orientation of the substrate? To address these questions, we used high-speed videography to record landing behavior of houseflies (*Musca domestica*) on substrates that were either vertical (*wall landings*) or inverted (*ceiling landings*). Using these data, we tested the hypothesis that body-deceleration and leg-extension responses are mutually coordinated, regardless of substrate orientation. If flying insects begin leg-extension at a fixed distance from the substrate, we expect less inter-trial variability in this distance at the onset of leg-extension. Tethered flight studies in houseflies indicate that onset of deceleration and leg-extension are correlated [19], implying that similar visual cues initiate both responses, *albeit* with different latencies. If so, we expect fixed time difference between the onset of deceleration and leg-extension.

## Materials and methods

Wild-caught adult houseflies (*Musca domestica*) were stored in a container with ad libitum access to sucrose and water. Because the flies were wild-caught, their precise age was indeterminate. In natural conditions, houseflies typically fly, maneuver and land at ambient illuminations ranging from 10^2^ (indoors or overcast outdoors) to 10^5^ Lux (sunny outdoors). All experiments described here were carried out within this range of illumination.

### Experimental setup and protocol

#### Wall landings

**Fig 1:**
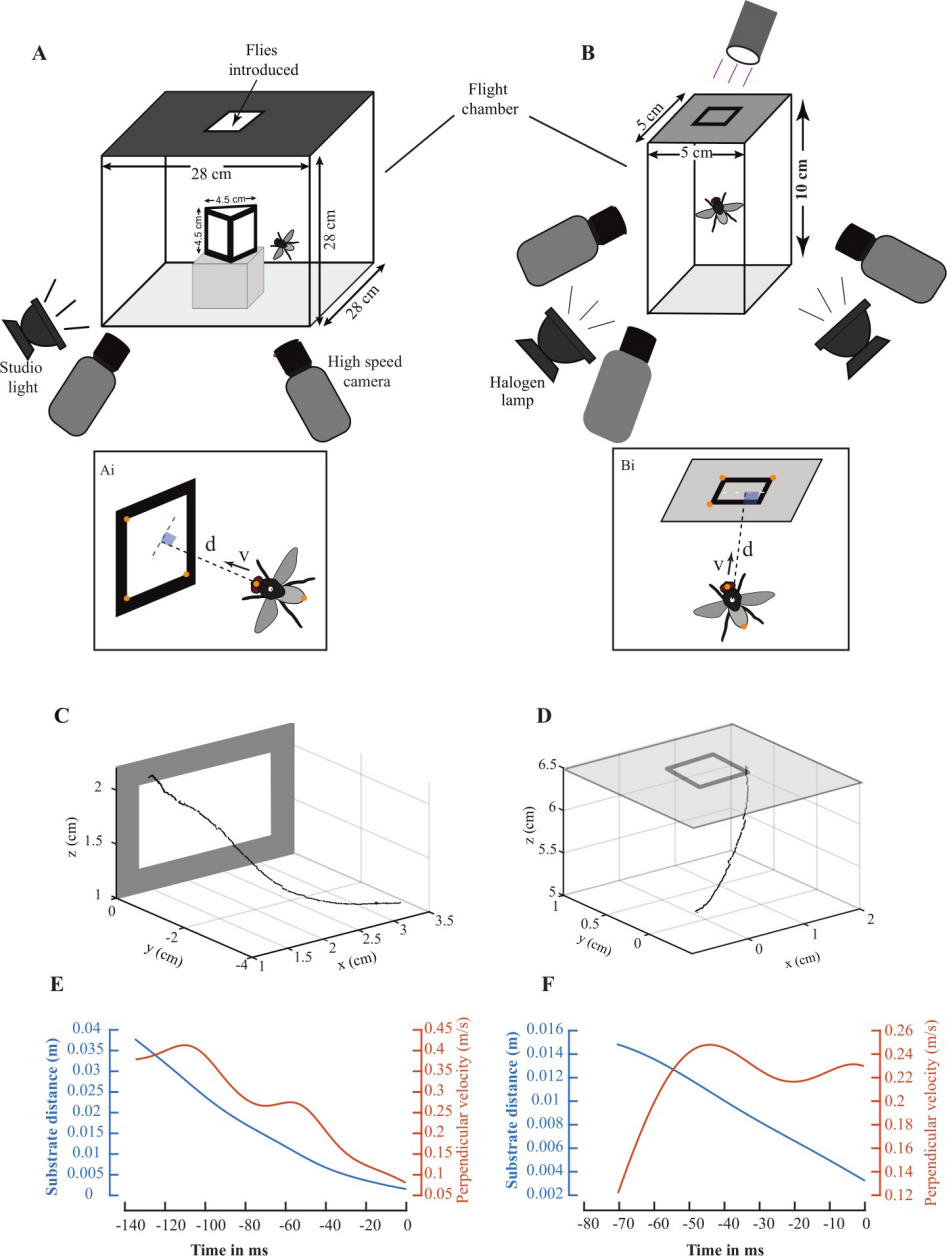
Experimental setups to record wall and ceiling landings, and measurement of the associated flight variables. Experimental setup for filming (A) wall landings elicited on a prism-shaped object and recorded using two synchronized high-speed cameras at 3000 fps, and (B) ceiling landings on a translucent ceiling recorded by three synchronized high-speed cameras at 4000 fps. For both vertical (Ai) and ceiling landings (Bi), we digitized tips of head and abdomen of fly in each frame, and three points on the landing substrate, and computed the midpoint of the line joining head and abdomen tips, and the distance of the midpoint from substrate (d). Flight velocity perpendicular to the plane of substrate (v) was computed using Equation 1. (C-D) Sample raw trajectories of the midpoint of a fly performing a vertical (C) and ceiling landing (D). (E-F) Below each trajectory, the substrate distance (blue) and perpendicular velocity (orange) are plotted against time-to-landing. Flies contacted the landing surface at 0 ms.

To film wall landings, we constructed a flight chamber comprised of a transparent plexiglass box (28 cm × 28 cm × 28 cm). At the center of this chamber, we placed an equilateral (each face 4.5 cm X 4.5 cm) prism-shaped object, lined with black edges, that served as landing substrate for flies. The chamber was lit by a studio lights (~3000 lux; Simpex Compact 300, Simpex Industries, Delhi, India). We introduced flies from the top of the filming chamber, and recorded their landings using two calibrated synchronized high-speed cameras (3000 fps; Phantom v7.3, Vision Research, Wayne, NJ, USA; Fig 1A, Ai). Flies typically performed a saccade towards the object before landing, similar to *Drosophila melanogaster* (van Breugel and Dickinson, 2012). The frame in which the saccade ended was selected as the start-point of each video, and the frame of first contact with substrate as the end-point.

#### Ceiling landings

To film ceiling landings, we constructed a smaller rectangular glass flight chamber (5 cm × 5 cm × 10 cm). One end of the chamber was lined with translucent filter paper (Fig 1B) and served as the ceiling. A black square outline (side length = 1.5 cm, line thickness = 2 mm) at the center of the ceiling provided an expansion stimulus for the approaching fly. A batch of 3–6 flies were starved for 10–12 hours, anesthetized using a 2.5 min cold shock (-20°C) and placed in the filming chamber. The chamber was illuminated by a UV torch placed above the ceiling to attract flies, two stereomicroscope lights (Nikon SMZ25; Nikon Corporation, Tokyo, Japan), and two 150 W halogen lamps (~30000 lux; Center 337 light meter, Center Technology Corporation, Taipei, Taiwan). Anesthetized flies recovered for 10–15 minutes. Ceiling landings were recorded using three calibrated, synchronized high-speed cameras at 4000 fps (2 Phantom v7.3/1 Phantom v611; Vision Research Inc., Ametek; Fig 1B, Bi). We recorded only one landing per batch of flies to avoid pseudo-replication. In most trials, flies took off from a lateral wall, rotated about their longitudinal axis (roll) by almost 360°, before ascending towards the ceiling. The video frame in which roll rotation ended was chosen as the start-point and the frame of first contact with substrate as end-point of each video.

### Digitization and computation of flight variables

To digitize videos of landings, we used MATLAB software by Hedrick (2008) (Mathworks, Natick, MA, USA). We digitized the tips of head and abdomen, and three points on the landing surface (Fig 1Ai and 1Bi). Multiple studies suggest that optic flow on the retina is the primary cue for triggering the behaviors associated with landing (such as deceleration, and leg-extension) [10,14,15,17–20,22,24]. The visual latency for pursuit in *Musca domestica* is 40±15 (μ±σ) ms, corresponding to temporal frequencies in the range of 15–40 Hz [25]. Additionally, tethered *Drosophila melanogaster* presented with expansion and rotational stimuli at temporal frequencies greater than 30 Hz do not exhibit a detectable optomotor response [26]. In accordance with these findings, we filtered the time series of the digitized points using a 4^th^ order Butterworth filter with cut-off frequency 30 Hz, because temporal fluctuations faster than 30 Hz are unlikely to be triggered by visual cues on the landing substrate. To ensure that our results are not biased by our choice of cut-off frequency of 30 Hz, we also used a second cut-off frequency of 40 Hz, corresponding to temporal fluctuations with a period of 25 ms, close to the fastest known visual reaction times of *Musca domestica* [25] (S2–S5 Figs).

Before applying the filter, we extrapolated the ends of time series data using quadratic functions to reduce edge effects [27]. We computed coordinates of the midpoint of the line joining head and abdomen tips (henceforth “midpoint”) at each frame to determine the broad trajectories during landing (Fig 1C and 1D). Two flight variables were computed from digitized points: First, perpendicular (shortest) distance of the midpoint from the substrate (d) and second, the component of flight velocity perpendicular to the plane of substrate (v)

$$v_{i}=\frac{d_{i‐1}‐d_{i+1}}{T}$$

in which i denotes the frame number, and T the time interval between (i-1) and (i+1) frames (2/3 ms for wall and 1/2 ms for ceiling landings (Fig 1E and 1F)).

#### Onset of deceleration

**Fig 2:**
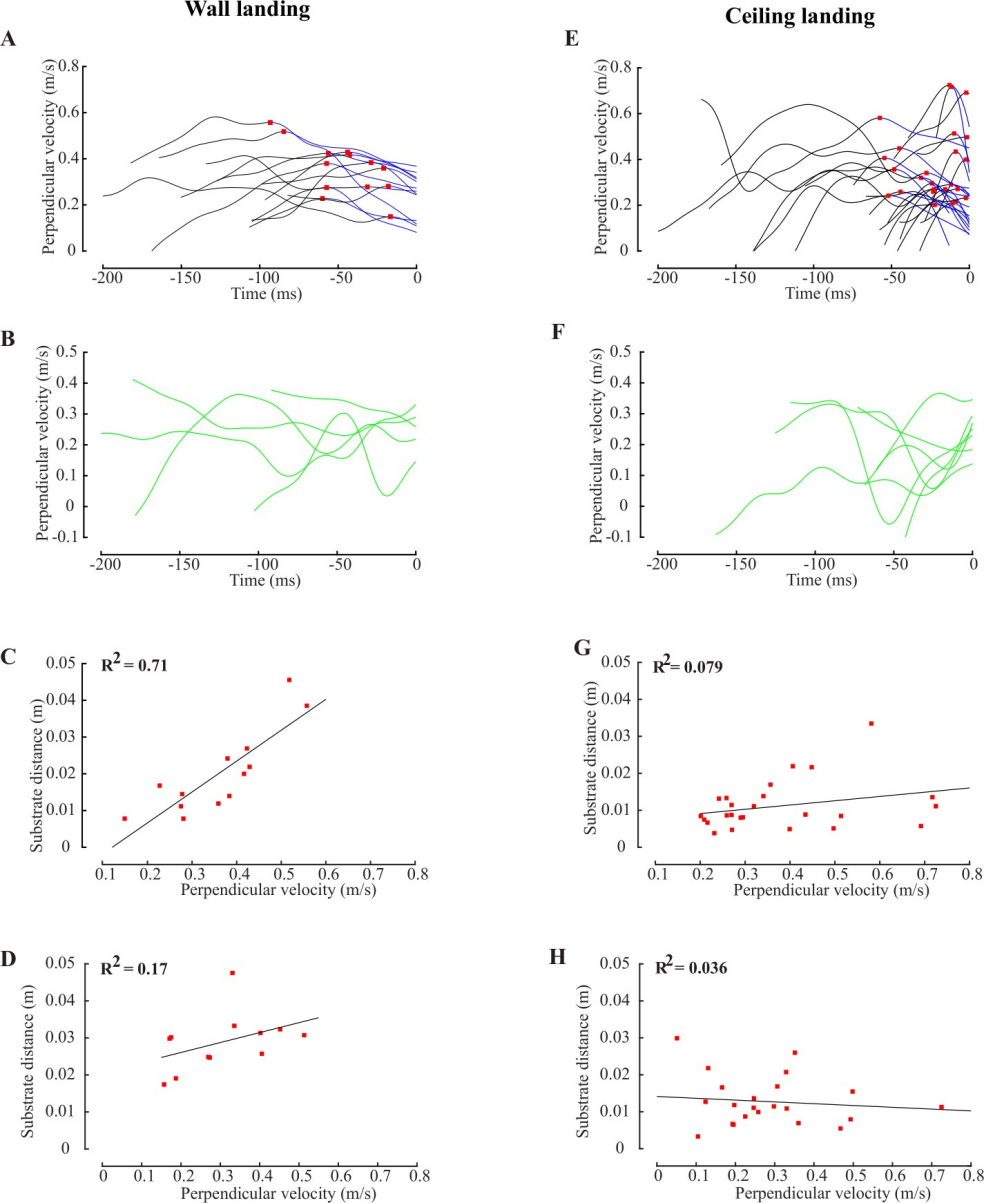
Initiation of deceleration and leg-extension during wall and ceiling landings. (A) Perpendicular velocity versus time-to-collision for all wall landings in which flies initiated deceleration before touchdown (n = 13). We identified the onset of deceleration (red squares, see Materials and methods) and decelerating segments of the flight trajectory (blue traces). (B) Perpendicular velocity versus time-to-collision for wall landings in which flies did not decelerate before touchdown (n = 5). (C) Substrate distance versus perpendicular velocity at the onset of deceleration for the 13 wall landings. Coefficient of determination (R^2^) of the best-fit line is 0.71. (D) Substrate distance versus perpendicular velocity at onset of leg-extension for 12 wall landings in which onset of leg-extension could be identified (see Materials and methods; R^2^ = 0.17). (E) Perpendicular velocity versus time for all ceiling landing trials in which flies decelerated before ceiling landing (n = 25). (F) Perpendicular velocity versus time for ceiling landings in which flies did not decelerate before touchdown (n = 7). (G) Substrate distance versus perpendicular velocity at the onset of deceleration for 25 ceiling landing trials (R^2^ = 0.079). (H) Substrate distance versus perpendicular velocity at onset of leg-extension for 22 ceiling landing trials in which flies extended their legs while approaching the substrate (but not during take-off, see Materials and methods; R^2^ = 0.036).

We wrote custom code in MATLAB to identify the local maxima/minima in plots of perpendicular velocity (v) *vs* time (Fig 2A, 2B, 2E and 2F; S2 Fig) and substrate distance as a function of perpendicular velocity (Fig 2C, 2D, 2G and 2H; S2 Fig). Trials in which the final extremum before touchdown was a minimum were classified as having no deceleration before landing (Fig 2B and 2F). In the remaining trials, final maximum velocity before first contact with the substrate was classified as *onset of deceleration* (Fig 2A and 2E; S2 Fig).

#### Onset of leg-extension

*Onset of leg-extension* was visually determined by close examination of videos and marking frames in which either one or both forelegs began to be raised. In 6 of 18 wall landings, the fly had extended its legs before arriving in the field of view of both cameras. Therefore, we could not determine the frame of onset of leg-extension for these trials. In 10 of 32 ceiling landing trials, flies extended their legs at takeoff but kept them extended. Because leg-extension was not elicited during landing in these trials, they were excluded from our analysis.

#### Testing hypotheses for the initiation of body-deceleration and leg-extension

**Fig 3:**
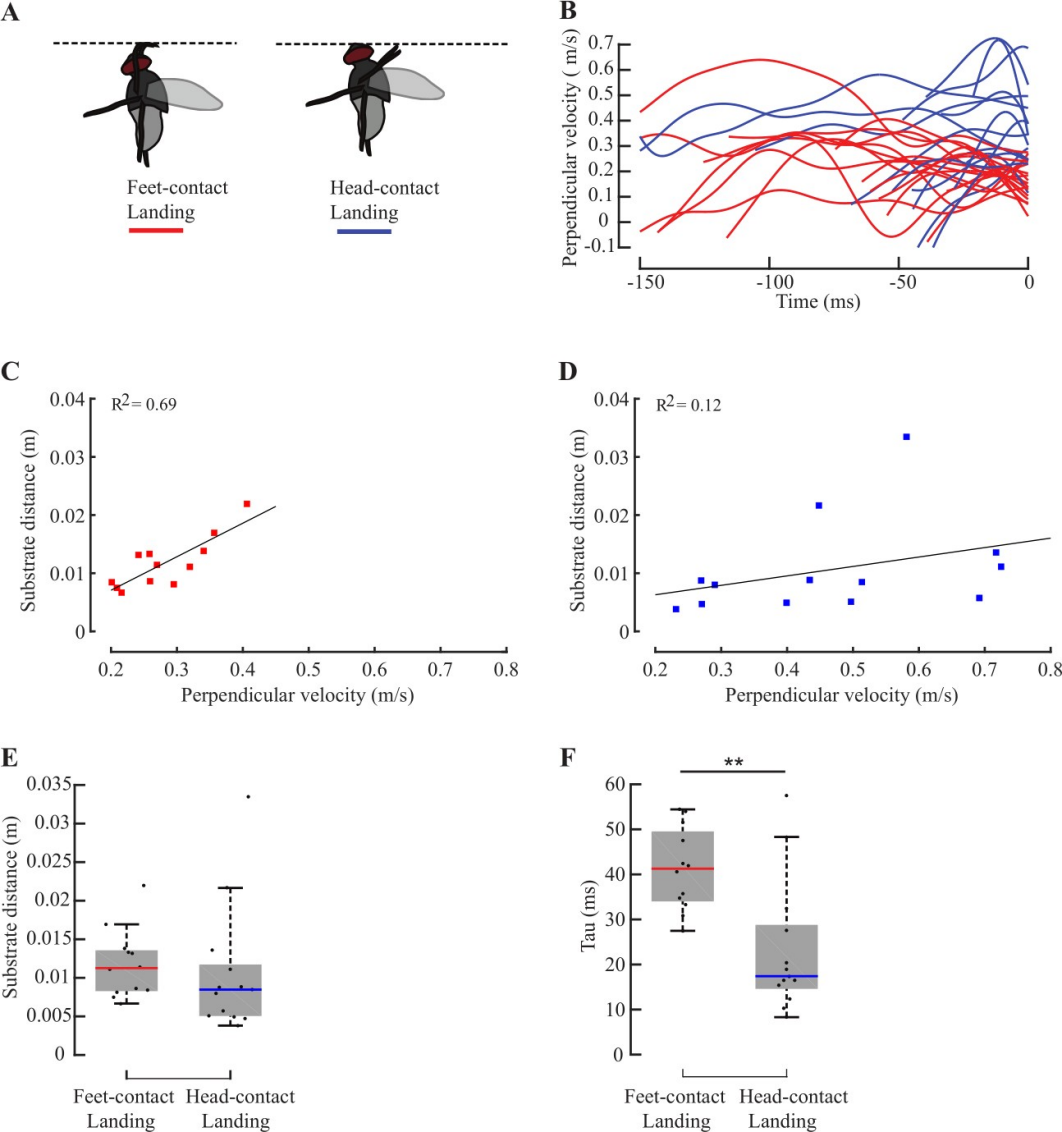
Onset of deceleration for flies performing feet-contact and head-contact ceiling landings. (A) Ceiling landings were grouped into head-contact landings (n = 15, blue) vs. feet-contact landings (n = 17, red) (B) Perpendicular velocity versus time-to-collision for all trials (n = 32) (C-D) 25 out of 32 flies decelerated before landing (see Materials and methods), and were analyzed further. Of these 25 ceiling landings, 12 performed a feet-contact landing and 13 flies executed a head-contact landing. (C) Substrate distance versus perpendicular velocity at the onset of deceleration for inverted feet-contact landings (n = 12, R^2^ = 0.69). (D) Substrate distance versus perpendicular velocity at the onset of deceleration for inverted head-contact landings (n = 13, R^2^ = 0.12). (E-F) Box plots for (E) substrate distance, and (F) *tau*, at the onset of deceleration for feet-contact and head-contact landings (Grey boxes indicate the central 50% data around the median (center line)). Whiskers represent 1.5 times interquartile range. Outliers were included in the analysis. Asterisks represent statistically different comparisons (*, **, ***, and **** represent p<0.05, p<0.01, p<0.001, p<0.0001 respectively). These conventions are used in all subsequent figures.

**Fig 4:**
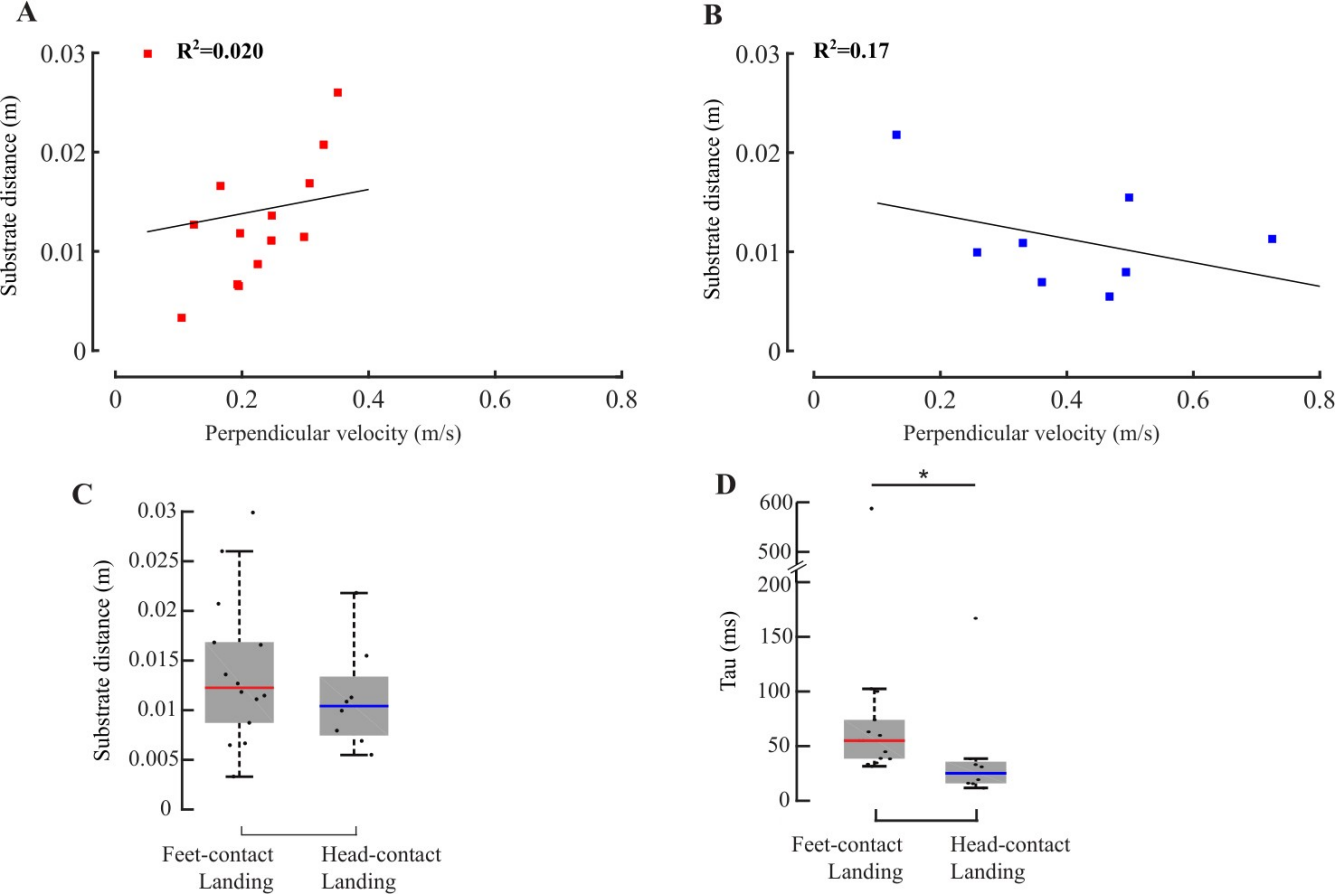
Onset of leg-extension for feet-contact and head-contact ceiling landings. Of the 22 flies which extended their legs when during ceiling landing (see Materials and methods), 14 executed a feet-contact and 8 a head-contact landing. (A) Substrate distance versus perpendicular velocity at the onset of leg-extension for feet-contact landings (n = 14; R^2^ = 0.020). (B) Substrate distance versus perpendicular velocity at onset of leg-extension for head-contact ceiling landings (n = 8, R^2^ = 0.17). (C-D) Box plots for (C) substrate distance, and (D) *tau*, at the onset of leg-extension for feet-contact and head-contact landings.

Do flies initiate both components of landing behavior at distances proportional to perpendicular velocity (*constant tau hypothesis*)? To address this, we plotted substrate distance (*d*) against perpendicular velocity (*v*) at the onset of deceleration (Fig 2C and 2G; Fig 3C and 3D; S2 Fig; S3 Fig) and leg-extension (Fig 2D and 2H; Fig 4A and 4B; S2 Fig; S4 Fig), and computed the coefficient of determination (R^2^) of the best-fit line using in-built MATLAB functions. The slope of this line is defined as *tau*. High R^2^ values would support the *constant tau hypothesis*.

**Fig 5:**
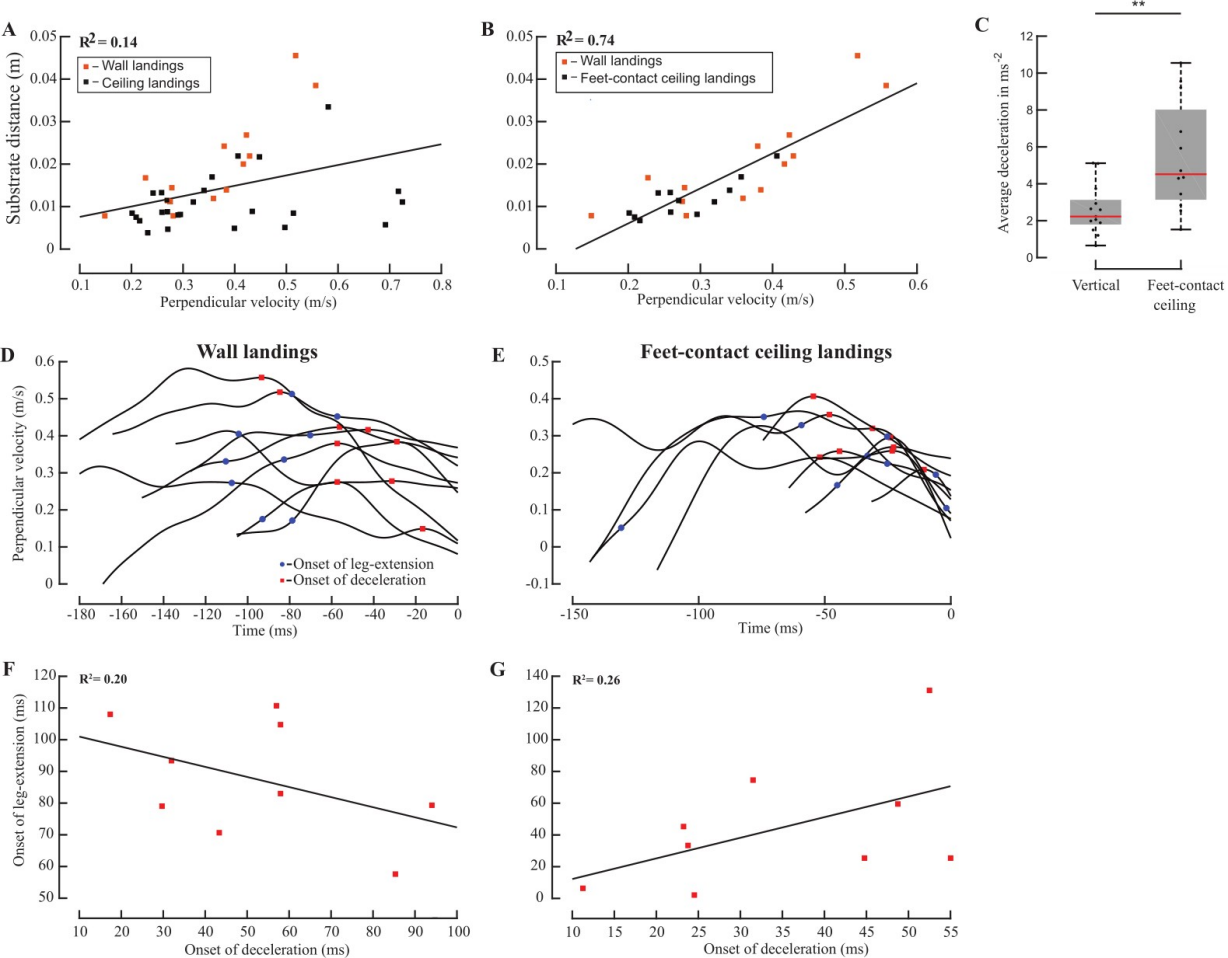
Comparing the onset of deceleration of wall and ceiling landings, and testing for correlation between the onsets of deceleration and leg-extension. (A) Substrate distance versus perpendicular velocity at the onset of deceleration for vertical (orange squares, n = 13) and ceiling landings (black squares, n = 25, R^2^ = 0.14). (B) Substrate distance versus perpendicular velocity at the onset of deceleration for vertical (n = 13) and feet-contact inverted (n = 12) landings (R^2^ = 0.74). (C) Flies performing feet-contact landings on inverted substrates decelerated at significantly higher rates compared to flies landing on vertical substrates (Wilcoxon ranksum test, p<0.01). (D, E) Perpendicular velocity vs. time for all trials (n = 9) in which onsets of deceleration (red squares) and leg-extension (blue circles) were known for (D) wall and (E) feet-contact ceiling landings. (F, G) Time-to-collision at onset of leg-extension versus time-to-collision at the onset of deceleration for (F) all 9 wall landings depicted in (D), and (G) all 9 feet-contact landings depicted in (E). Low R^2^ imply weak correlation.

The above data allowed us to test two predictions. First, if flies initiated a behavior at a fixed substrate distance, then the inter-trial variability of this distance should be low. Second, if the same cues elicited both body-deceleration and leg-extension but with different latencies, then time difference between the modules should be conserved. Hence, we plotted time-to-collision at the onset of leg-extension (duration between onset of leg-extension and contact with substrate) against time-to-collision at the onset of deceleration (duration between onset of deceleration and contact with the substrate; Fig 5F and 5G). High R^2^-values would support the hypothesis that both modules were elicited by the same stimuli.

### Statistical tests

As we cannot *a priori* assume normal distribution of the data on substrate distance and *tau* values, we used non-parametric Wilcoxon rank sum test to compare various quantities (Fig 3E and 3F; Fig 4C and 4D; coded in MATLAB).

## Results

### Initiation of deceleration and leg-extension

#### Wall landings

Prior to landing on vertical surfaces, flies decelerate their body and extend their legs. Of the 18 wall landing trials, we observed deceleration before touchdown in 13 trials (Fig 2A). In the remaining 5 trials (Fig 2B), flies did not decelerate but we observed leg-extension (See Materials and methods). In cases with clear deceleration phase, we observed a strong linear relationship between substrate distance and perpendicular velocity at onset of deceleration (coefficient of determination (R^2^) = 0.71; Fig 2C). Such flies typically approached vertical walls at velocities ranging between 0.1–0.55 m/s. These data support the constant-*tau* hypothesis for onset of deceleration. However, correlation between substrate distance and perpendicular velocity at onset of leg-extension was weaker (R^2^ = 0.17; Fig 2D), suggesting that leg-extension is not initiated at a threshold *tau*.

#### Ceiling landings

Of 32 flies which performed ceiling landings, 25 decelerated before landing (Fig 2E), but 7 did not despite leg-extension (Fig 2F). Similar to wall landings, these flies also approached the ceiling at velocities less than 0.4 m/s. For flies that decelerated, observed only a weak linear relationship between substrate distance and perpendicular velocity at onset of deceleration (R^2^ = 0.079; Fig 2G) and leg-extension (R^2^ = 0.036; Fig 2H). Thus, for ceiling landings, neither deceleration nor leg-extension were initiated at threshold *tau*.

Ceiling landings may be grouped into two categories. In 15 trials, flies bumped their head during landing (henceforth *head-contact landing*), whereas in the remaining 17, only the feet touched the substrate during landing (*feet-contact landing*; Fig 3A). Head-contacts are symptomatic of lack of control. In *head-contact landing*, flies displayed body-deceleration, leg-extension(See supplementary videos) in response to an approaching surface. However, these flies typically approached inverted substrates at greater speeds (blue lines, Fig 3B) than in *feet-contact landing* (red lines, Fig 3B). Note that all flies first contacted the landing surface with their tarsi, even in *head-contact landings* (See supplementary videos).

Of the 25 flies which decelerated before landing, 12 performed *feet-contact landing* and 13 performed *head-contact landing* (Fig 3B). In *feet-contact landings*, substrate distance varied linearly with perpendicular velocity at onset of deceleration (n = 12; R^2^ = 0.69; Fig 3C); thus, these flies initiated deceleration at fixed *tau*. However, in *head-contact landings*, substrate distance at which deceleration was initiated was weakly related to perpendicular velocity (R^2^ = 0.12; Fig 3D); thus, if flies did not decelerate at or before threshold *tau*, they failed to land in a controlled manner. Thus, flies must initiate deceleration at a specific *tau* before controlled (*feet-contact*) vertical or ceiling landing (Figs 2C and 3C). In contrast to body-deceleration, correlation between substrate distance and perpendicular velocity at onset of leg-extension was weak, regardless of substrate orientation (vertical; Fig 2D; inverted; Fig 2H). Thus, the deceleration module may be elicited independently of leg-extension module.

Are the approach kinematics related to control of landing? Although the flies performing head-contact landings initiated deceleration at similar distances as those who contacted the substrate with their feet (Wilcoxon ranksum test, p>0.05; Fig 3E), their *tau* values significantly differed (Wilcoxon ranksum test, p<0.01; Fig 3F). In *feet-contact landings*, substrate distance at onset of deceleration increased linearly with perpendicular velocity (constant *tau*), and *tau* values were greater at onset of deceleration as compared to head-contact. This suggests that an optimal *tau* margin of 41±9 ms (μ±σ) was required for initiating deceleration in controlled landings; flies that missed this window were likely to collide their heads against the ceiling. Flies performing *feet-contact* as well as *head-contact* landings decelerated at similar rates (Wilcoxon Ranksum Test, p>0.05; S1 Fig), suggesting that flies missing the *tau* margin did not decelerate faster to compensate.

Of 22 ceiling landings in which the flies initiated leg-extension during flight (but not during take-off, Materials and methods), 14 executed *feet-contact landings* and 8 executed *head-contact ceiling landings*. The relationship between substrate distance and perpendicular velocity at onset of leg-extension was weak for both *feet-contact* (n = 14; R^2^ = 0.020; Fig 4A) and *head-contact landings* (n = 8; R^2^ = 0.17; Fig 4B). Thus, in *feet-contact landings* on inverted substrates, flies did not initiate leg-extension at constant *tau*. In fact, substrate distance at which flies initiated leg-extension in a *head-contact landing* was not significantly different from *feet-contact landings* (Wilcoxon ranksum test, p>0.05; Fig 4C) but they initiated leg-extension at significantly lower *tau* (Wilcoxon ranksum test, p<0.05; Fig 4D). Thus, longer *tau* is essential for controlled landing.

### Dependence of onset of deceleration on substrate orientation

Substrate distance is only weakly correlated with perpendicular velocity at onset of deceleration for both vertical (orange) and ceiling landings (black; Fig 5A) (R^2^ = 0.14), if we also include data for *head-contact landings*. Excluding the *head-contact landings*, however, reveals stronger correlation between substrate distance and perpendicular velocity at onset of deceleration (R^2^ = 0.74). Thus, in feet-contact landings, flies initiate deceleration at similar *tau* on both vertical or inverted surfaces (Fig 5B). Additionally, distributions of *tau* at onset of deceleration was not significantly different between wall and ceiling landings (Wilcoxon ranksum test, p>0.5; S1 Fig), suggesting that similar neuronal and mechanistic processes initiated the onset of deceleration in both cases. Of all flies that land feet-first on the substrate, the deceleration of those approaching the vertical substrate was lower than those approaching an inverted substrate (Wilcoxon ranksum test, p<0.01; Fig 5C). Thus, their deceleration depends on substrate orientation.

### Correlation between deceleration and leg-extension

Although flies initiated deceleration in specific range of *tau* before smoothly landing on vertical or inverted substrates (Figs 2C and 3C), the correlation between distance and perpendicular velocity at onset of leg-extension was weak, regardless of substrate orientation (Figs 2D, 2H, 4A and 4B), indicating that each module is elicited by different cues. In 9 of 18 wall landings and 9 of 17 feet-contact landings, flies initiated deceleration and leg-extension when flying towards the substrate (Materials and methods). If both modules are initiated by the same set of stimuli, then we expect a clear correlation in the order and time difference between the onsets of each module. Flies initiated leg-extension *before* deceleration in 7 out of 9 wall landings (Fig 5D), and in 4 out of 9 ceiling landings (Fig 5E). The correlation between time-to-collision at onset of leg-extension and time-to-collision at onset of deceleration was weak for both vertical (R^2^ = 0.20; Fig 5F) and feet-contact landings (R^2^ = 0.26; Fig 5G). It is thus unlikely that deceleration and leg-extension were elicited by the same set of cues.

## Discussion

We filmed houseflies *Musca domestica* landing on wall and inverted horizontal surfaces. While landing on vertical surfaces, houseflies initiated deceleration at distances proportional to flight velocity component normal to landing surface i.e. fixed *tau* (Fig 2C). Nearly half the flies bumped their head on the substrate while landing on inverted substrates (see Supplementary videos) whereas the rest touched their tarsi, before swiveling and landing. In the feet-contact ceiling landings, deceleration was initiated at a threshold value of *tau* (Fig 3C), similar to wall landings (Fig 5B). The correlation between substrate distance and perpendicular velocity at onset of leg-extension was weak regardless of substrate orientation (vertical or inverted; Fig 2D and 2H), or type of landing (feet- or head-contact) (Figs 2H, 4A and 4B). Flies that bumped their head on the substrate during ceiling landings typically approached the substrate at higher velocities (Fig 3B). Additionally, they triggered deceleration (Fig 3F) and leg-extension (Fig 4D) at lower *tau* compared to feet-contact landings.

### Similarities between wall and ceiling landings

Ceiling landings require flies to approach the substrate from below, whereas in wall landings, the substrate is approached from all directions. Hence, the experimental setups used for studying wall and ceiling landings (Materials and methods, Fig 1A and 1B) were different. Ceiling landings were filmed in a more constrained, collimated chamber than the one used for filming wall landings. The visual cues that elicited landing in flies on vertical (a white square (side = 4.5 cm outlined with 0.5 cm wide black stripe) vs. inverted substrates (square outline side = 1.5 cm, thickness = 2 mm) were also different. Additionally, wall landings were filmed at lower light intensities (~3000 lux, overcast day), whereas ceiling landings at intensities closer to sunlight (~30000 lux). Both lighting conditions were well within the range experienced by houseflies in their natural habitats.

Despite these differences, flies initiated deceleration at similar *tau* before landing in a controlled manner on both wall and inverted substrates. The final moments of the wall landing maneuver are highly stereotypic: flies always pitch-up before contacting the landing surface (see Supplementary videos). Because horizontal velocities of houseflies [28] and *Drosophila melanogaster* [29] are inversely correlated with the pitch angle, flies approaching vertical substrates likely induce deceleration by increasing their body pitch. However, ceiling landings were more variable. Such landings involved pitch-up maneuvers before landing in some cases, but a combination of roll, pitch and yaw maneuvers before landing in others (see Supplementary videos). Despite variability in ceiling landings, flies that performed *feet-contact ceiling landings* initiated deceleration at a constant *tau* values. Thus, cues that trigger deceleration in landing flies are independent of orientation of the landing surface.

### Dependence of the results on the cut-off frequency of the Butterworth filter

Prior to computation of distances, and velocities, we filtered the time series of the position of the fly using a 4^th^ order Butterworth filter, cut-off frequency being 30 Hz. Temporal fluctuations faster than 30 Hz are unlikely to be visual responses to cues on the landing surface [25,26]. To ensure that our major conclusions are not an artifact of the choice of cut-off frequency, we also filtered the time series of positions at 40 Hz, corresponding to the fastest known visual reactions in *Musca domestica* of 25 ms [25]. We measured correlations between distance from the substrate and perpendicular velocity at the onsets of both deceleration and leg-extension (see S2–S5 Figs). Our conclusions remain the same, houseflies initiate deceleration at constant *tau* before landing *feet-contact* on the vertical or inverted surface (S2 Fig; S3 Fig; S5 Fig). However, flies begin to extend their legs at a distance independent of flight velocity normal to the landing substrate (S2 Fig; S4 Fig).

### Variability and versatility of landing responses

Whereas houseflies approaching the vertical surface primarily undergo a pitch-up maneuver before touchdown, those approaching inverted surfaces may rotate about all three axes. 15 of 32 flies landing on the ceiling bumped their head on the substrate while landing. These flies typically approached the ceiling at greater velocity (Fig 3B), and initiated body-deceleration and leg-extension at lower values of *tau* (Figs 3F and 4D). We did not observe such collisions in flies landing on vertical surfaces, perhaps due to the different experimental conditions. Flies performing ceiling landings were anesthetized *via* a brief cold shock prior to filming, whereas the flies performing wall landings were released in the chamber without a cold shock. Because cold shock is known to affect some insect behaviors (e.g. courtship in *Drosophila melanogaster* [30]), we cannot rule out the possibility that it affected the landing performance of houseflies. However, we have observed cold-anesthetized houseflies routinely perform chases and territorial behaviors that require rapid responses, suggesting that their flight recovers substantially from the cold-shock treatment, after a period of recovery. On the other hand, collisions may be a normal part of the landing behavior, especially for ceiling landings. Collisions with the substrate have also been documented in previous papers. For instance, around 36% of *Drosophila melanogaster* approaching a cylindrical landing post collided with it [8]. In these experiments, the sub-population that collided did not differ from landing flies in the retinal size-dependent threshold velocity at which they initiated body-deceleration. Instead, these flies decelerated at lower rates, often failing to extend their legs before touchdown.

In the current study, we found no significant differences in the rates of body-deceleration between *feet-contact* vs. *head-contact landings* (Wilcoxon ranksum test, p>0.05; S1 Fig). Also, in *head-contact landings*, flies extended their legs but had lower *tau* than *feet-contact* landings (Fig 3D). Because we filmed a single ceiling landing from a batch of 4–6 flies, we could not ascertain whether a sub-population of flies performed poorly during ceiling landings. 5 out of 18 flies in wall landings, and 7 out of 32 flies in ceiling landings did not initiate deceleration before landing, perhaps because they did not experience sufficiently low values of *tau*. Another recent study demonstrated that *Drosophila melanogaster* decelerate to a near hover state, followed by acceleration before landing on a vertical pole [31]. In our study, houseflies usually decelerated continuously until landing (Fig 2A and 2E), highlighting the variation in visual control of deceleration across flying insects. The biomechanics of landing maneuvers also contributes greatly to their deceleration profile.

For both wall and ceiling landings, flies initiated leg-extension at a point that was independent of substrate distance and perpendicular velocity. In 10 out of 32 ceiling landings, flies initiated leg-extension during takeoff, implying that either leg-extension is not tightly regulated, or is sensitive to finer cues including size, velocity, and contrast of an approaching object [10,19]. Additionally, sudden changes in light intensity [10,14] or front-to-back optic flow [14–18] also elicit leg-extension in tethered flies.

Thus, the *tau*-dominated body-deceleration but variable leg-extension together contribute to both the stereotypy and versatility in landing behaviors of flies.

### Computation of *tau* by flies

Houseflies approaching spherical substrates are known to initiate deceleration at threshold *tau* values [9]. However, flies landing on a sphere can potentially contact its surface at any inclination ranging from horizontal to inverted, which was not recorded in the study. Our data show that houseflies initiate deceleration at fixed values of *tau* regardless of whether they land feet-first on vertical or inverted substrates. According to the retinal size-dependent threshold model, which explains onset of deceleration in *Drosophila melanogaster* approaching a cylindrical surface [8], flies estimate *tau* from optic flow, and initiate deceleration when *tau* falls below a threshold. The results of this study were, however, experimentally indistinguishable from the constant *tau* model.

Our study adds to the growing body of evidence that animal nervous systems compute *tau* and use it to control multiple behaviors. For example, birds approaching a target maintain the rate of change of *tau* (*taudot*) at constant values, resulting in characteristic deceleration profiles [5,6]. Pigeons approaching a perch begin leg-extension at fixed *tau* [6]. Gannets plunge diving into the sea begin streamlining when *tau* reduces below a threshold [32]. Bees approaching a surface maintain *tau* at a constant value, resulting in a proportionate decrease in flight velocity with distance [4,7]. How do flies estimate *tau* from optic flow? When insects approach a substrate, the instantaneous *tau* is approximately equal to the ratio of angular separation between two points on the surface, and rate of change of angular separation between these points (if they are close; [33]). Thus, to estimate *tau*, the nervous system must compute angular size, and rate of angular expansion of objects and compare them in real time. Despite numerous behavioral examples of *tau* estimation in animals, studies demonstrating neural computation of *tau* are scarce. We know of only one example of computation of a threshold *tau* value by a neuron in pigeons [34,35], in which response onset and peak firing to a looming object of a sub-population of neurons in nucleus rotundus occurred at fixed *tau*, irrespective of angular size or object velocity.

*Tau* can be measured by comparing the rate of expansion and angular size of a moving stimulus. Are there examples of neurons or neuronal clusters which measure either of these quantities in insects? A recent study in bees showed that descending neurons in the ventral nerve cord monotonically increased their median firing rate with angular velocity of a frontally-presented rotating spiral stimulus, up to a specific angular velocity beyond which the response saturated. However, median response of the neurons was also a function of the number of arms in the rotating spiral (which correlates with spatial frequency) [36]. In flies, lobula plate tangential cells (LPTCs) integrate inputs from local motion detectors and respond to wide-field motion (for a detailed review see [37]). A subset of LPTCs called horizontal system (HS) cells respond to horizontal optic flow [38] generated by moving gratings of varying contrast, wavelength, and velocity [39]. However, the HS cells of a hoverflies presented with moving naturalistic images, reliably encoded their angular velocity with little dependence on their contrast [40]. Examples of neurons which measure angular size of a looming object are also reported in bullfrogs [41], pigeons [35], and locusts [42,43]. It is thus likely that certain neurons in houseflies estimate angular size and angular expansion in the visual neuropil. So far, no study has demonstrated neurons which compute the ratio of angular size to angular expansion in insects. A vast majority of the studies of neuronal response to visual stimuli document the firing properties of neurons in the brain or the ventral nerve cord. It is possible that angular expansion and angular size are compared by interneurons in the thoracic ganglia. Simultaneous presentation of looming stimuli and single unit recordings from the thoracic ganglia are required to test this hypothesis.

## Supporting information

S1 Fig Average deceleration in feet-contact vs head-contact ceiling landings and comparison of *tau* at onset of deceleration between wall and feet-contact ceiling landings. (A) Before landing, there was no significant difference (Wilcoxon ranksum test, p>0.05) in rate of deceleration between feet-contact and head-contact landings. (B) There was no significant difference in *tau* at the onset of deceleration between wall and feet-contact ceiling landings (Wilcoxon ranksum test, p>0.05).
(TIF)

S2 Fig Initiation of deceleration and leg-extension during wall and ceiling landings, when the cut-off frequency for the Butterworth filter is increased to 40 Hz. (A) Perpendicular velocity versus time-to-collision for all wall landings in which flies initiated deceleration before touchdown (n = 14). We identified the onset of deceleration (red squares, see Materials and methods) and decelerating segments of the flight trajectory (blue traces). (B) Perpendicular velocity versus time-to-collision for wall landings in which flies did not decelerate before touchdown (n = 4). (C) Substrate distance versus perpendicular velocity at the onset of deceleration for the 14 wall landings. Coefficient of determination (R^2^) of the best-fit line is 0.64. (D) Substrate distance versus perpendicular velocity at onset of leg-extension for 12 wall landings in which onset of leg-extension could be identified (see Materials and methods; R^2^ = 0.17). (E) Perpendicular velocity versus time for all ceiling landing trials in which flies decelerated before ceiling landing (n = 24). (F) Perpendicular velocity versus time for ceiling landings in which flies did not decelerate before touchdown (n = 8). (G) Substrate distance versus perpendicular velocity at the onset of deceleration for 25 ceiling landing trials (R^2^ = 0.20). (H) Substrate distance versus perpendicular velocity at onset of leg-extension for 22 ceiling landing trials in which flies extended their legs while approaching the substrate (but not during take-off, see Materials and methods; R^2^ = 0.010).
(TIF)

S3 Fig Onset of deceleration for flies performing feet-contact and head-contact ceiling landings, when the cut-off frequency for the Butterworth filter is increased to 40 Hz. (A-B) 24 out of 32 flies decelerated before landing (see Materials and methods), and were analyzed further. Of these 24 ceiling landings, 13 performed a feet-contact landing and 11 flies executed a head-contact landing. (A) Substrate distance versus perpendicular velocity at the onset of deceleration for inverted feet-contact landings (n = 13, R^2^ = 0.69). (D) Substrate distance versus perpendicular velocity at the onset of deceleration for inverted head-contact landings (n = 11, R^2^ = 0.15).
(TIF)

S4 Fig Onset of leg-extension for feet-contact and head-contact ceiling landings, when the cut-off frequency for the Butterworth filter is increased to 40 Hz. Of the 22 flies which extended their legs when during ceiling landing (see Materials and methods), 14 executed a feet-contact and 8 a head-contact landing. (A) Substrate distance versus perpendicular velocity at the onset of leg-extension for feet-contact landings (n = 14; R^2^ = 0.020). (B) Substrate distance versus perpendicular velocity at onset of leg-extension for head-contact ceiling landings (n = 8, R^2^ = 0.15).
(TIF)

S5 Fig Comparing the onset of deceleration of wall and ceiling landings, when the cut-off frequency for the Butterworth filter is increased to 40 Hz. (A) Substrate distance versus perpendicular velocity at the onset of deceleration for vertical (orange squares, n = 14) and ceiling landings (black squares, n = 24, R^2^ = 0.26). (B) Substrate distance versus perpendicular velocity at the onset of deceleration for vertical (n = 14) and feet-contact inverted (n = 13) landings (R^2^ = 0.65).
(TIF)

S1 Movie Wall landing. (AVI)

S2 Movie Feet-contact ceiling landing. (AVI)

S3 Movie Head-contact ceiling landing. (AVI)

S4 Movie Ceiling landing: Fly pitches up before landing. (AVI)

S5 Movie Ceiling landing: Fly rolls before landing. (AVI)

S6 Movie Ceiling landing: Fly yaws, pitches and rolls before landing. (AVI)

## References

[1] Evangelista C, Kraft P, Dacke M, Reinhard J, Srinivasan MV. The moment before touchdown: landing manoeuvres of the honeybee Apis mellifera. J Exp Biol. 2010;213: 262–270. 10.1242/jeb.037465 20038660

[2] Hyzer WG. Flight behavior of a fly alighting on a ceiling. Science. 1962;137: 609–610. 10.1126/science.137.3530.609 17836544

[3] Reber T, Baird E, Dacke M. The final moments of landing in bumblebees, Bombus terrestris. J Comp Physiol A. 2016;202: 277–285. 10.1007/s00359-016-1073-4 26868924

[4] Baird E, Boeddeker N, Ibbotson MR, Srinivasan MV. A universal strategy for visually guided landing. Proc Natl Acad Sci. 2013;110: 18686–18691. 10.1073/pnas.1314311110 24167269 PMC3831993

[5] Lee DN, Reddish PE, Rand DT. Aerial docking by hummingbirds. Naturwissenschaften. 1991;78: 526–527.

[6] Lee DN, Davies MN, Green PR. Visual control of velocity of approach by pigeons when landing. J Exp Biol. 1993;180: 85–104.

[7] Srinivasan MV, Zhang S-W, Chahl JS, Barth E, Venkatesh S. How honeybees make grazing landings on flat surfaces. Biol Cybern. 2000;83: 171–183. 10.1007/s004220000162 11007294

[8] Van Breugel F, Dickinson MH. The visual control of landing and obstacle avoidance in the fruit fly Drosophila melanogaster. J Exp Biol. 2012;215: 1783–1798. 10.1242/jeb.066498 22573757

[9] Wagner H. Flow-field variables trigger landing in flies. Nature. 1982;297: 147.

[10] Goodman L. J. (1960). The landing responses of insects: I. The landing response of the fly, Lucilia sericata, and other Calliphorinae. Journal of Experimental Biology, 37(4), 854–878.

[11] Reber T, Dacke M, Warrant E, Baird E. Bumblebees perform well-controlled landings in dim light. Front Behav Neurosci. 2016;10: 174 10.3389/fnbeh.2016.00174 27683546 PMC5021987

[12] Zhao J, Huang H, Yan S. Honey bees (*Apis mellifera ligustica*) swing abdomen to dissipate residual flying energy landing on a wall. J Appl Phys. 2017;121: 094702 10.1063/1.4977844

[13] Lee DN. The optic flow field: The foundation of vision. Phil Trans R Soc Lond B. 1980;290: 169–179. 6106236 10.1098/rstb.1980.0089

[14] Borst A. Time course of the houseflies’ landing response. Biol Cybern. 1986;54: 379–383.

[15] Borst A. Temporal processing of excitatory and inhibitory motion stimuli in the fly’s landing system. Sci Nat. 1989;76: 531–534.

[16] Borst A, Bahde S. Comparison between the movement detection systems underlying the optomotor and the landing response in the housefly. Biol Cybern. 1987;56: 217–224.

[17] Borst A, Bahde S. Visual information processing in the fly’s landing system. J Comp Physiol A. 1988;163: 167–173.

[18] Borst A, Bahde S. What kind of movement detector is triggering the landing response of the housefly? Biol Cybern. 1986;55: 59–69.

[19] Borst A, Bahde S. Spatio-temporal integration of motion. Naturwissenschaften. 1988;75: 265–267.

[20] Borst A. How Do Flies Land? BioScience. 1990;40: 292–299. 10.2307/1311266

[21] Coggshall JC. The landing response and visual processing in the milkweed bug, Oncopeltus fasciatus. J Exp Biol. 1972;57: 401–413.

[22] De Talens AFP, Ferreti CT. Landing reaction of Musca domestica: dependence on dimensions and position of the stimulus. J Exp Biol. 1970;52: 233–256. 5442285 10.1242/jeb.52.2.233

[23] Eckert H. Orientation sensitivity of the visual movement detection system activating the landing response of the blowflies, Calliphora, and Phaenicia: A behavioural investigation. Biol Cybern. 1980;37: 235–247.

[24] Tammero LF, Dickinson MH. Collision-avoidance and landing responses are mediated by separate pathways in the fruit fly, Drosophila melanogaster. J Exp Biol. 2002;205: 2785–2798. 12177144 10.1242/jeb.205.18.2785

[25] Srinivasan MV, Bernard GD. The pursuit response of the housefly and its interaction with the optomotor response. J Comp Physiol A. 1977;115: 101–117. 10.1007/BF00667788

[26] Duistermars BJ, Chow DM, Condro M, Frye MA. The spatial, temporal and contrast properties of expansion and rotation flight optomotor responses in Drosophila. J Exp Biol. 2007;210: 3218–3227. 10.1242/jeb.007807 17766299

[27] Walker JA. Estimating velocities and accelerations of animal locomotion: a simulation experiment comparing numerical differentiation algorithms. J Exp Biol. 1998;201: 981–995.

[28] Wagner H. Flight performance and visual control of flight of the free-flying housefly (Musca domestica L.) I. Organization of the flight motor. Phil Trans R Soc Lond B. 1986;312: 527–551.

[29] David CT. The relationship between body angle and flight speed in free-flying Drosophila. Physiol Entomol. 1978;3: 191–195.

[30] Barron AB. Anaesthetising Drosophila for behavioural studies. J Insect Physiol. 2000;46: 439–442. 10.1016/S0022-1910(99)00129-8 12770207

[31] Shen C, Sun M. Wing and body kinematics measurement and force analyses of landing in fruit flies. Bioinspir Biomim. 2017;13: 016004 10.1088/1748-3190/aa934b 29027521

[32] Lee DN, Reddish PE. Plummeting gannets: a paradigm of ecological optics. Nature. 1981;293: 293.

[33] Lee DN. A theory of visual control of braking based on information about time-to-collision. Perception. 1976;5: 437–459. 10.1068/p050437 1005020

[34] Wang Y, Frost BJ. Time to collision is signalled by neurons in the nucleus rotundus of pigeons. Nature. 1992;356: 236–238. 10.1038/356236a0 1552942

[35] Sun H, Frost BJ. Computation of different optical variables of looming objects in pigeon nucleus rotundus neurons. Nat Neurosci. 1998;1: 296–303. 10.1038/1110 10195163

[36] Ibbotson MR, Hung Y-S, Meffin H, Boeddeker N, Srinivasan MV. Neural basis of forward flight control and landing in honeybees. Sci Rep. 2017;7 10.1038/s41598-017-14954-0 29109404 PMC5673959

[37] Borst A, Haag J, Reiff DF. Fly motion vision. Annu Rev Neurosci. 2010;33: 49–70. 10.1146/annurev-neuro-060909-153155 20225934

[38] Hausen K. Motion sensitive interneurons in the optomotor system of the fly. Biol Cybern. 1982;45: 143–156. 10.1007/BF00335241

[39] Egelhaaf M, Borst A. Transient and steady-state response properties of movement detectors. JOSA A. 1989;6: 116–127. 10.1364/josaa.6.000116 2921651

[40] Straw AD, Rainsford T, O’Carroll DC. Contrast sensitivity of insect motion detectors to natural images. J Vis. 2008;8: 32 10.1167/8.3.32 18484838

[41] Nakagawa H, Hongjian K. Collision-sensitive neurons in the optic tectum of the bullfrog, Rana catesbeiana. J Neurophysiol. 2010;104: 2487–2499. 10.1152/jn.01055.2009 20810689

[42] Gabbiani F, Krapp HG, Laurent G. Computation of object approach by a wide-field, motion-sensitive neuron. J Neurosci. 1999;19: 1122–1141. 9920674 10.1523/JNEUROSCI.19-03-01122.1999 PMC6782150

[43] Gabbiani F, Mo C, Laurent G. Invariance of angular threshold computation in a wide-field looming-sensitive neuron. J Neurosci. 2001;21: 314–329. 11150349 10.1523/JNEUROSCI.21-01-00314.2001 PMC6762430

