# Supplementary figures and images for: Landing maneuvers of houseflies on vertical and inverted surfaces
Source: PLoS One. 2019 Aug 14;14(8):e0219861. doi: 10.1371/journal.pone.0219861 (PMC6693754; doi:10.1371/journal.pone.0219861)

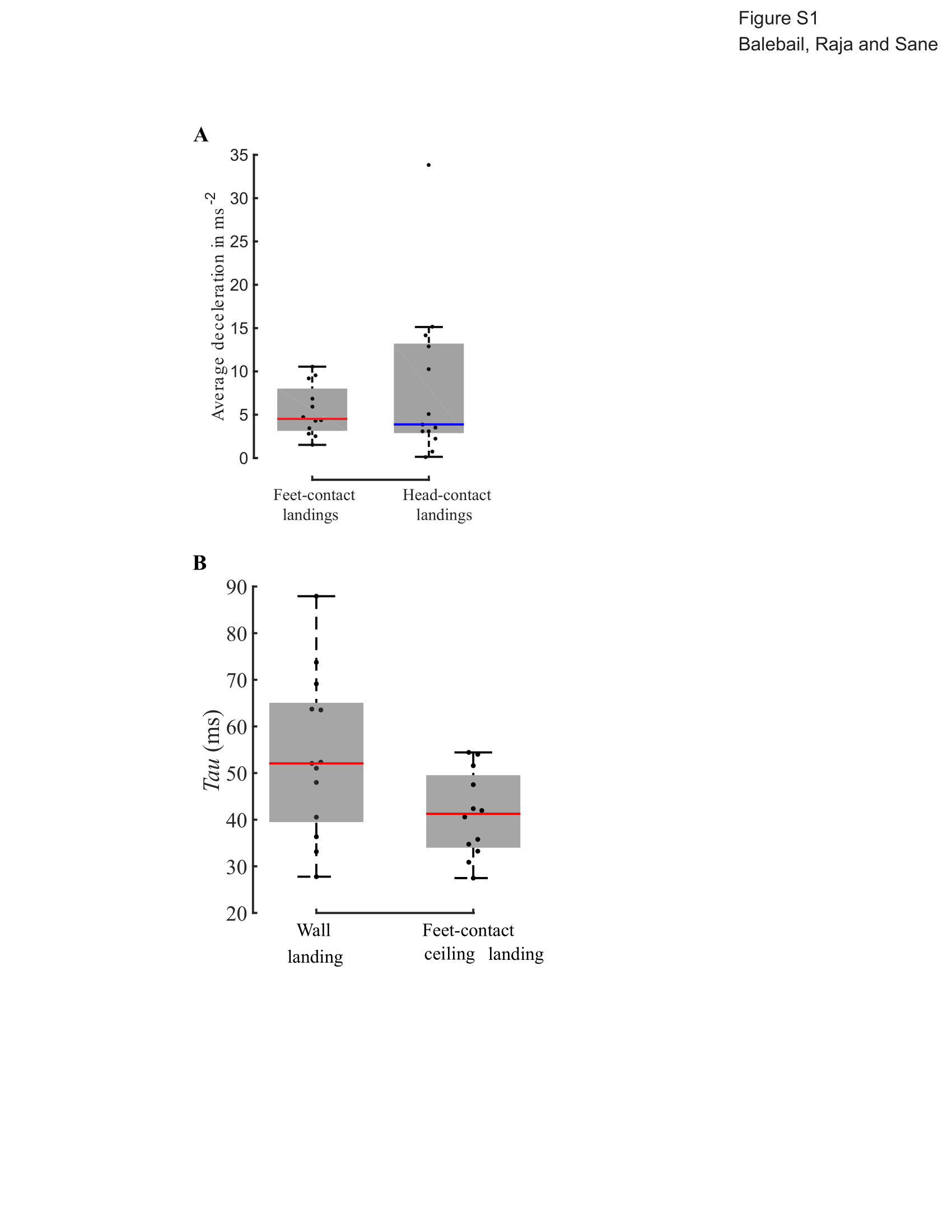

Supplement: S1 Fig — (A) Before landing, there was no significant difference (Wilcoxon ranksum test, p>0.05) in rate of deceleration between feet-contact and head-contact landings. (B) There was no significant difference in tau at the onset of deceleration between wall and feet-contact ceiling landings (Wilcoxon ranksum test, p>0.05). (TIF) [file pone.0219861.s001.tif]

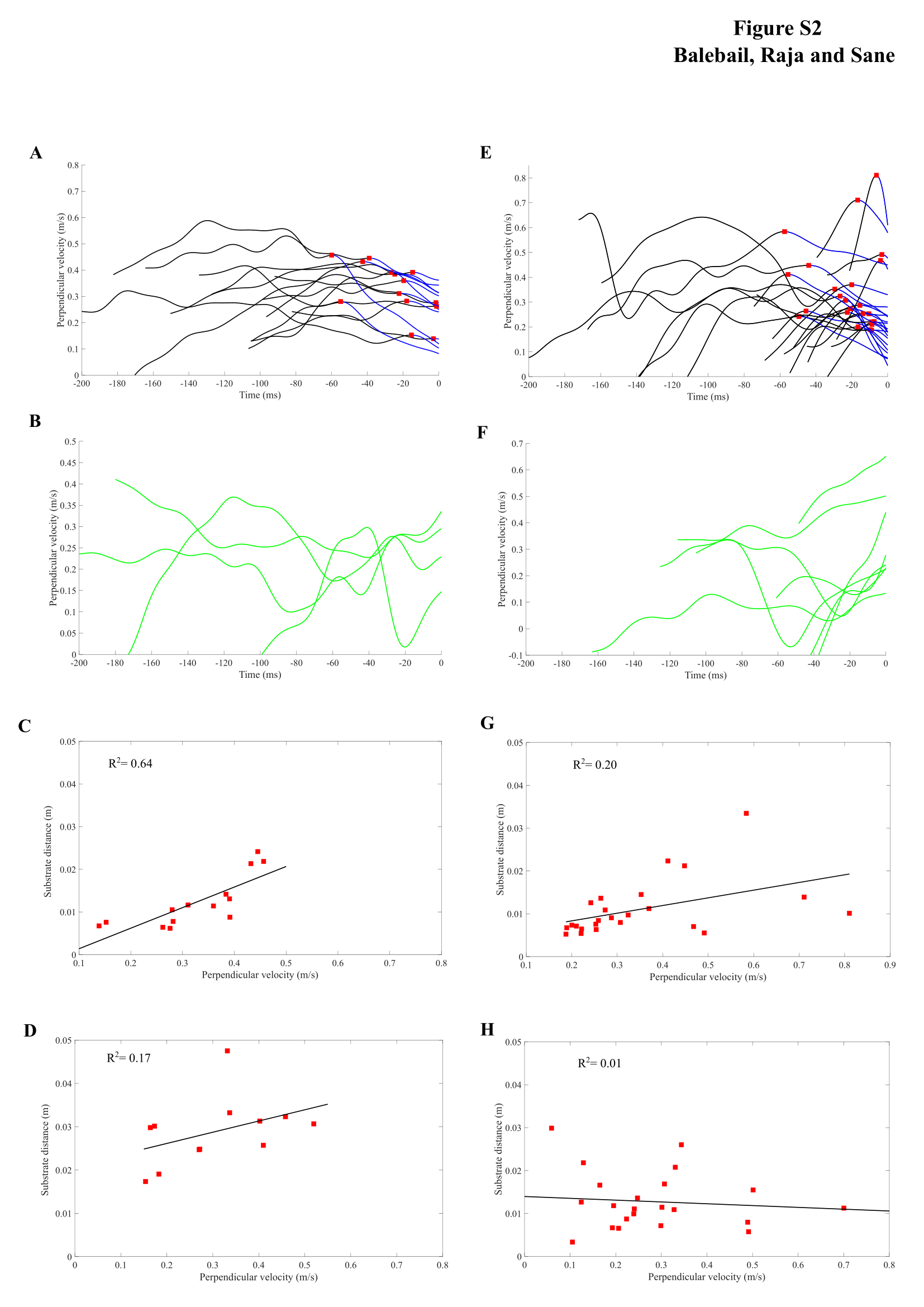

Supplement: S2 Fig — (A) Perpendicular velocity versus time-to-collision for all wall landings in which flies initiated deceleration before touchdown (n = 14). We identified the onset of deceleration (red squares, see Materials and methods) and decelerating segments of the flight trajectory (blue traces). (B) Perpendicular velocity versus time-to-collision for wall landings in which flies did not decelerate before touchdown (n = 4). (C) Substrate distance versus perpendicular velocity at the onset of deceleration for the 14 wall landings. Coefficient of determination (R2) of the best-fit line is 0.64. (D) Substrate distance versus perpendicular velocity at onset of leg-extension for 12 wall landings in which onset of leg-extension could be identified (see Materials and methods; R2 = 0.17). (E) Perpendicular velocity versus time for all ceiling landing trials in which flies decelerated before ceiling landing (n = 24). (F) Perpendicular velocity versus time for ceiling landings in which flies did not decelerate before touchdown (n = 8). (G) Substrate distance versus perpendicular velocity at the onset of deceleration for 25 ceiling landing trials (R2 = 0.20). (H) Substrate distance versus perpendicular velocity at onset of leg-extension for 22 ceiling landing trials in which flies extended their legs while approaching the substrate (but not during take-off, see Materials and methods; R2 = 0.010). (TIF) [file pone.0219861.s002.tif]

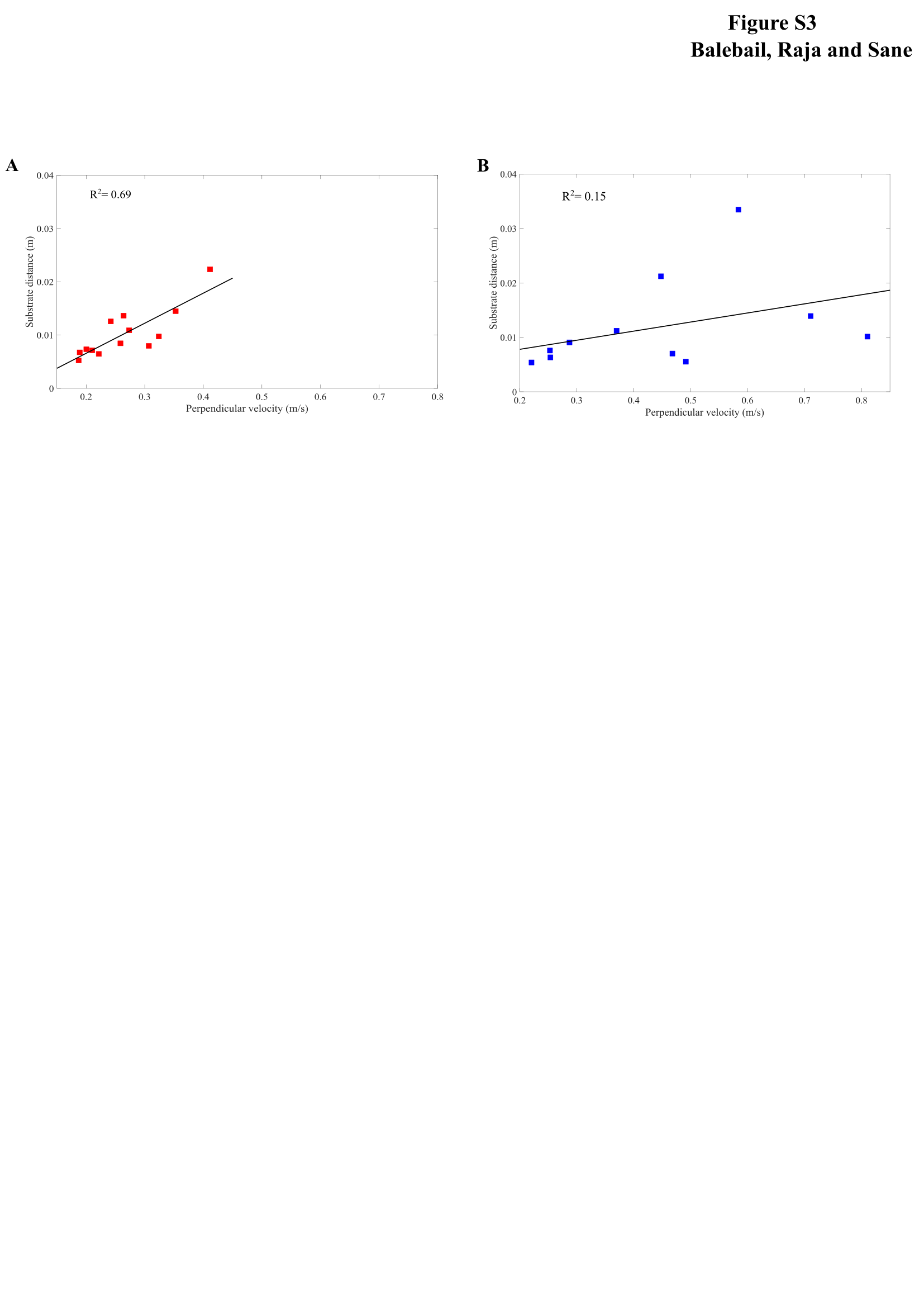

Supplement: S3 Fig — (A-B) 24 out of 32 flies decelerated before landing (see Materials and methods), and were analyzed further. Of these 24 ceiling landings, 13 performed a feet-contact landing and 11 flies executed a head-contact landing. (A) Substrate distance versus perpendicular velocity at the onset of deceleration for inverted feet-contact landings (n = 13, R2 = 0.69). (D) Substrate distance versus perpendicular velocity at the onset of deceleration for inverted head-contact landings (n = 11, R2 = 0.15). (TIF) [file pone.0219861.s003.tif]

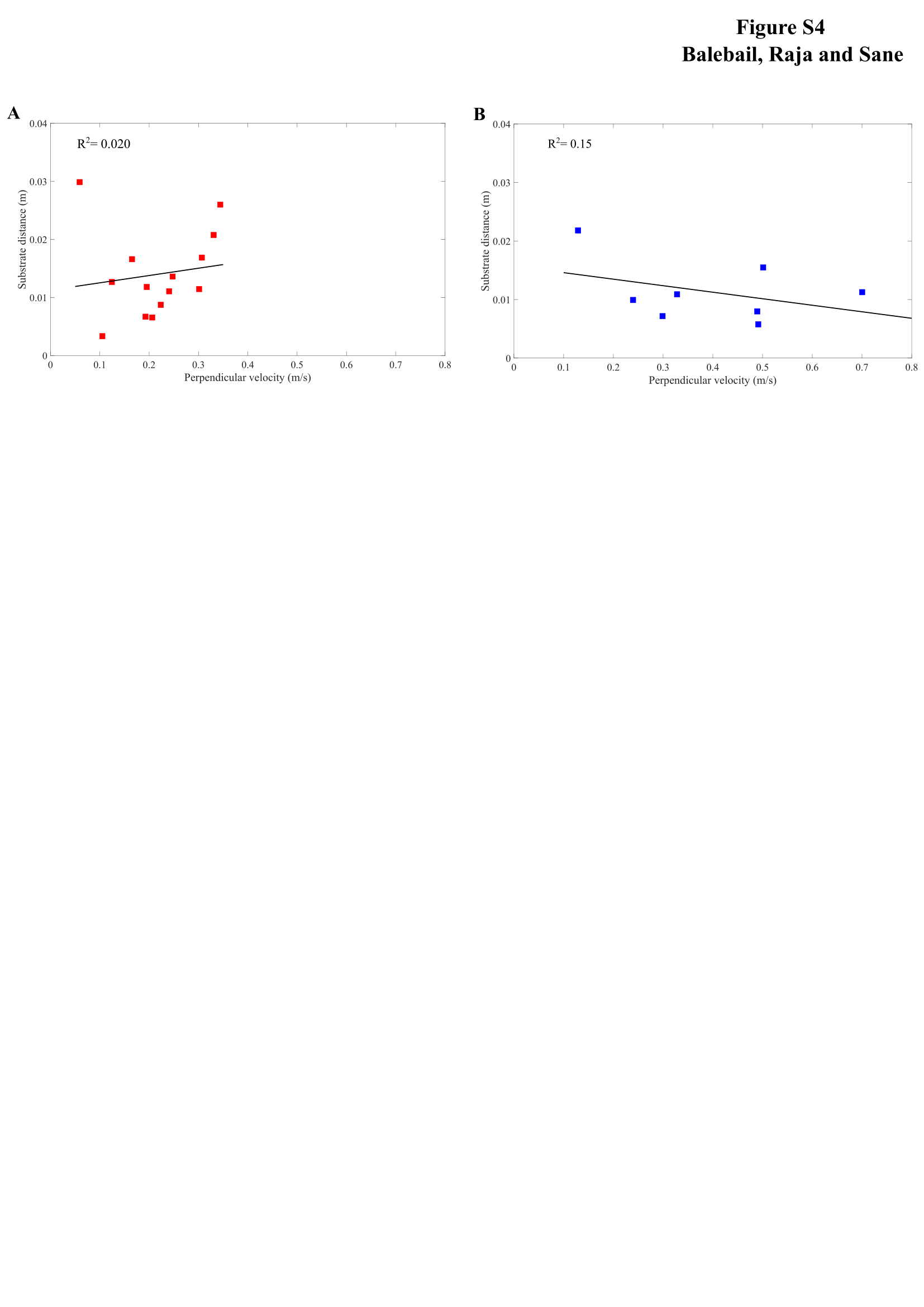

Supplement: S4 Fig — Of the 22 flies which extended their legs when during ceiling landing (see Materials and methods), 14 executed a feet-contact and 8 a head-contact landing. (A) Substrate distance versus perpendicular velocity at the onset of leg-extension for feet-contact landings (n = 14; R2 = 0.020). (B) Substrate distance versus perpendicular velocity at onset of leg-extension for head-contact ceiling landings (n = 8, R2 = 0.15). (TIF) [file pone.0219861.s004.tif]

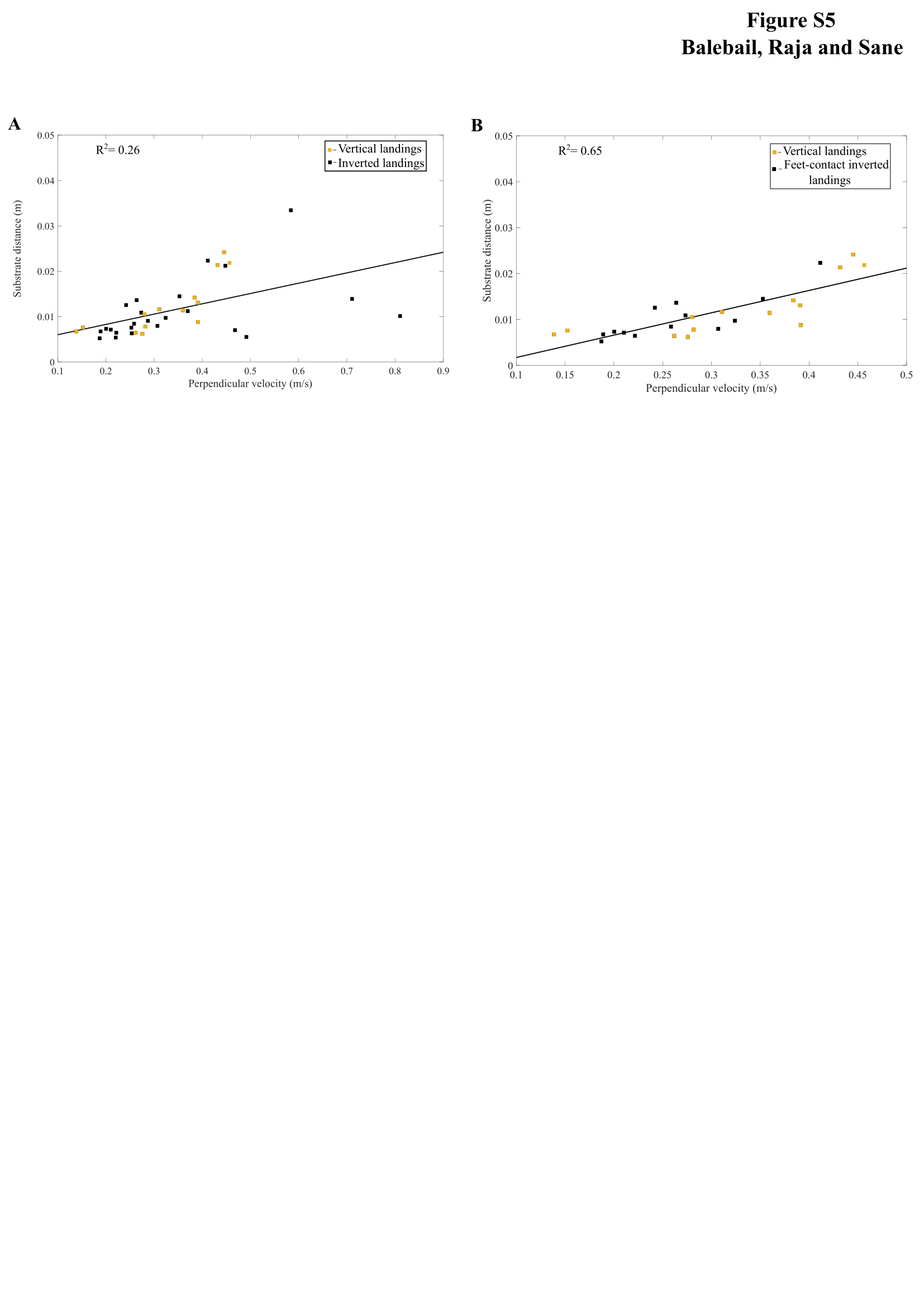

Supplement: S5 Fig — (A) Substrate distance versus perpendicular velocity at the onset of deceleration for vertical (orange squares, n = 14) and ceiling landings (black squares, n = 24, R2 = 0.26). (B) Substrate distance versus perpendicular velocity at the onset of deceleration for vertical (n = 14) and feet-contact inverted (n = 13) landings (R2 = 0.65). (TIF) [file pone.0219861.s005.tif]
